# Supplementary material for: Stepwise progression of β-selection during T cell development involves histone deacetylation
Source: Life Sci Alliance. 2022 Oct 25;6(1):e202201645. doi: 10.26508/lsa.202201645 (PMC9595210; doi:10.26508/lsa.202201645)
Supplement: Supplementary file 1 [file LSA-2022-01645_TableS1.docx]

Table S1 Antibody used in the study

| Antibody | Supplier | Clone/Catlog | Usage (titre) | |
| --- | --- | --- | --- | --- |
| CD11b | BD Biosciences | M1/70 | Flow cytometry | 1:1000 |
| NK1-1 | BD Biosciences | PK136 | Flow cytometry | 1:1000 |
| CD45R | Tonbo Biosciences | RA3-6B2 | Flow cytometry | 1:1000 |
| Ly6G.6C | BD Biosciences | RB6-8C5 | Flow cytometry | 1:1000 |
| TER119 | BioLegend | TER119 | Flow cytometry | 1:1000 |
| CD4 | BioLegend | RM4-5 | Flow cytometry | 1:400 |
| CD8 | BioLegend | 53-6.7 | Flow cytometry | 1:400 |
| CD44 | BioLegend | IM7 | Flow cytometry | 1:300 |
| CD25 | BioLegend | PC61 | Flow cytometry | 1:300 |
| CD28 | BioLegend | E18 | Flow cytometry | 1:200 |
| CD27 | BioLegend | LG.3A10 | Flow cytometry | 1:800 |
| CD2 | BioLegend | RM2-5 | Flow cytometry | 1:500 |
| CD71 | BioLegend | RI7217 | Flow cytometry | 1:400 |
| CD98 | BioLegend | 4F2 | Flow cytometry | 1:400 |
| CD5 | Tonbo Biosciences | 53-7.3 | Flow cytometry | 1:300 |
| TCRβ | Tonbo Biosciences | H57-597 | Flow cytometry | 1:200 |
| TCRγδ | BioLegend | GL3 | Flow cytometry | 1:400 |
| Ki-67 | BioLegend | 16A8 | Flow cytometry | 1:800 |
| c-Myc | Cell Signaling Technology | 5605 | Flow cytometry | 1:250 |
| Lef1 | Cell Signaling Technology | 2230 | Flow cytometry | 1:500 |
| Notch1 | Abcam | ab27526 | Flow cytometry | 1:300 |
|  |  |  | Immunofluorescence | 1:200 |
| LAT | Cell Signaling Technology | 9166 | Immunofluorescence | 1:200 |
| Ac-tubulin | Cell Signaling Technology | 5335 | Flow cytometry | 1:500 |
|  |  |  | Immunofluorescence | 1:300 |
| Ac-tubulin | Abcam | Ab24610 | Immunofluorescence | 1:1000 |
| H3K18ac | Abcam | Ab1191 | Flow cytometry | 1:1000 |
| HDAC6 | Merck | 07-732 | Flow cytometry | 1:500 |
| HDAC6 | Novus Biologicals | NBP1-78981 | Immunofluorescence | 1:500 |
